# Antioxidant Activities of Various Extracts from *Artemisisa selengensis* Turcz (LuHao) 

**DOI:** 10.3390/molecules15074934

**Published:** 2010-07-16

**Authors:** Feng Shi, Xiaobin Jia, Chenglei Zhao, Yan Chen

**Affiliations:** Key Laboratory of Delivery Systems of Chinese Meteria Medica, Jiangsu Provincial Academy of Chinese Medicine, Jiangsu, Nanjing, 210028,China; E-Mails: zhaochenglei@gmail.com (C.Z.); shifeng_1985_wcl@163.com (F.S.); ychen202@yahoo.com.cn (Y.C.)

**Keywords:** *Artemisia selegensis* Turcz, antioxidant activity, phenolic acids, flavonoids, SOD, MDA

## Abstract

The antioxidant activities of the various extracts and fractions from the herbs of *Artemisia selegensis* Turcz (AST) were investigated by *in vitro* and *in vivo* assays. FRAP, DPPH and ABTS assays were used to evaluate the antioxidant activities of the extracts/fractions *in vitro*. The effect of water extract (WE) in reducing oxidative stress in male mice was evaluated. Phenolic acid compounds contribute significantly to the antioxidant activity. From the results of three *in vitro* antioxidant assays, WE was found to have the highest antioxidant activity, and among the WE subfractions, the water soluble fraction has a significant antioxidant activity. The *in vivo* antioxidant assay results showed that high doses of WE significantly decrease the MDA level compared to normal diet and D-(+) galactose group (*p* < 0.05), and the SOD activity of mice given a high dose of WE was the highest. These *in vitro* and *in vivo* studies demonstrated that the extracts, especially the WE from AST, have significant antioxidant and free radical scavenging activities. In summary, we propose that AST could be potentially used as a rich source of natural antioxidants.

## 1. Introduction

In recent years, natural antioxidants, particularly those present in fruits and vegetables, have attracted increasing interest among consumers and the scientific community. Epidemiological studies have demonstrated that frequent intake of fruits and vegetables is associated with a lower risk of age-related disease such as coronary heart diseases [1] and cancer [2,3]. Many natural foodstuffs contain dietary antioxidants that can scavenge free radicals. Some studies have indicated that phenolic substances, such as flavonoids, phenolic acids and tannins are much more potent antioxidants than vitamin C and vitamin E [4]. These phenolic compounds also possess diverse biological functions including anti-inflammatory, anti-carcinogenic, and anti-atherosclerotic activities, which may be related to their antioxidant activity [5]. Other studies have shown a high degree of correlation between the total antioxidant activity of some fruits and their phenolic contents [6].

In addition to antioxidants present in fruits and vegetables, another important source of antioxidants is herbs, including those derived from Traditional Chinese Medicines (TCMs) [7,8], which may possess more potent antioxidant activity than common dietary plants.

*Artemisia selegensis* Turcz (AST, Luhao in Chinese) belongs to the Asteraceae Artemisia plant family; it is a well-known traditional Chinese medicine, and is also widely used as a health food product for its taste and nutritional properties [9]. AST was recorded by both Shennong's Herbolary (Shen Nong Ben Cao Jing) and the Compendium of Materia Medica (Ben Cao Gang Mu). AST is widely distributed in China, and had been used from ancient times to relieve fever, dysentery and acute or chronic hepatitis and has been shown to possess antioxidant activities. Previous research found that the pharmaceutical action of AST was closely related to some components in it such as phenolic acids, flavonoids and alkaloids [10,11]. However, it appears logical that the different methods of extraction could affect its antioxidant activities. We extracted AST using the traditional boiling water extraction method and a more modern extraction method using 70% ethanol, or 95% ethanol. Because the aqueous extract has a high antioxidant activity, it was further fractionated with different solvents. The antioxidant activities of the secondary extracts were evaluated using three *in vitro* methods: the Trolox equivalent antioxidant capacity (TEAC) assay with ABTS radical cation, the ferric reducing antioxidant power (FRAP) assay and the 2,2-diphenyl-1-picryldydrazyl (DPPH) assay. The total phenolic acid contents and the flavonoid contents of extracts were determined using the Folin-Ciocalteu method and the AlCl_3_ method, respectively.

The aim of this research was to compare the ability of FRAP, DPPH and ABTS assays to estimate antioxidant potentials of various extracts and correlations between antioxidant potentials and contents of flavonoids and total phenolic acids present in AST extracts. We also investigated the relationship between *in vitro* and *in vivo* antioxidant activity. The *in vivo* antioxidant activity of the water extract (WE) from AST was determined by monitoring its effect on antioxidant enzymes and the levels of malonaldehyde (MDA) in oxidative stress induced male mice.

## 2. Results and Discussion

### 2.1. Total phenolic acid and flavonoid contents

The amount of total phenolic acids and flavonoids differed significantly among the various AST extracts (Table 1). The values of phenolic acid contents varied from 8.0 to 557.3 mg GAE/100 g dry weight of plant material as measured by Folin-Ciocalteau method. The flavonoid contents values ranged from 11.7 to 987.0 mg rutin/100 g of dry plant material as measured by the AlCl_3_ method. The WE (water extract) was found to have the highest phenolic acid content value (557.3 mg GAE/100 g), followed by the 70% ethanol extract (311.0 mg GAE/100 g) and the 95% ethanol extract (165.2 mg GAE/100 g). However, the highest value of flavonoid content was determined in the 70% ethanol extract (987.0 mg rutin/100 g), followed by the WE (549.9 mg rutin/100 g) and the 95% ethanol extract (406.5 mg rutin/100 g). For the WE subfractions, the sequence of total phenolic acid content was: WT (water fraction of WE, 414.5 mg GAE/100 g) > EA (ethyl acetate fraction of WE, 39.6 mg GAE/100 g) > BU (*n*-butanol fraction of WE, 34.3 mg GAE/100 g) > PE (petroleum ether fraction of WE, 8.0 GAE/100 g), but for total flavonoids content it was: WT (223.9 mg rutin/100 g) > BU (76.9 mg rutin/100 g ) > EA(53.5 mg rutin/100 g) > PE(11.7 mg rutin/100 g). From above results we can see that the total phenolic acid contents were different from the total flavonoids in the various AST extracts.

**Table 1:** Total phenolic acid and flavonoid contents of the extracts and fractions from AST.

Extracts/fractions	Total phenolic acids^a^	Total flavonoids^b^
WE	557.3 ± 5.5	549.9 ± 3.6
70% ethanol extract	311.0 ± 3.1	987.0 ± 2.9
95% ethanol extract	165.2 ± 0.6	406.5 ± 4.1
PE	8.0 ± 0.6	11.7 ± 0.4
EA	39.6 ± 4.1	53.5 ± 1.2
BU	34.3 ± 0.6	76.9 ± 0.7
WT	414.5 ± 2.3	223.9 ± 5.5

^a^ Total phenolic acids content expressed in mg gallic acid equivalents/100 g dry weight of AST; ^b^ Total flavonoids content expressed in mg rutin equivalents/100 g dry weight of AST; Each value is the mean ± SD of triplicate measurements; WE: water extract; PE: petroleum ether fraction of WE; EA: ethyl acetate fraction of WE; BU: n-butanol fraction of WE; WT: water fraction of WE.

### 2.2. In vitro total antioxidant capacity of extracts from AST

#### 2.2.1. Total antioxidant power of extracts/fractions from AST by the FRAP assay

In this study we used the FRAP assay because it is quick and simple to perform and the reaction is reproducible and linearly related to the molar concentration of the antioxidants [12]. This method was initially developed to assay plasma antioxidant capacity, but can be used to measure the antioxidant capacity in a wide range of biological samples and pure compounds like fruits, wines, and animal tissues [13,14]. The FRAP can be readily applied to both water and ethanol extracts of different plants. In this assay, the antioxidant activity is determined on the basis of the ability to reduce ferric (III) iron to ferrous (II) iron. The results are expressed as μmol ferrous iron equivalents per 100 g of dry weight of plant material.

**Figure 1:**
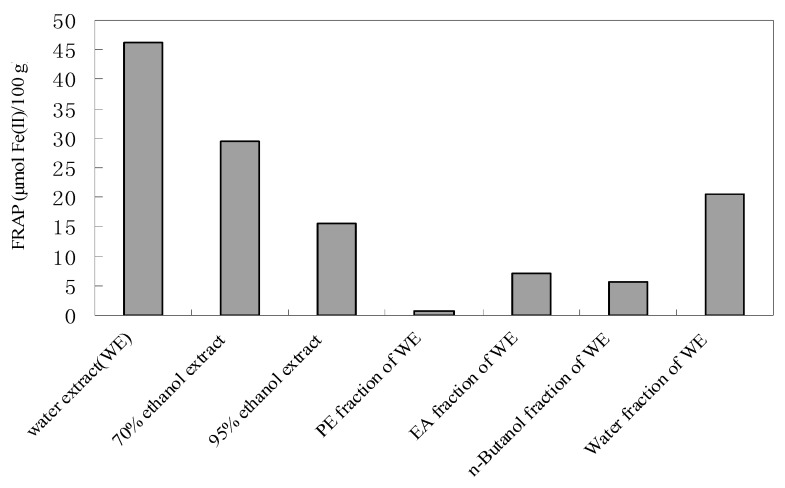
Antioxidant activity (μmol Fe(II)/100 g) of extracts from AST.

Figure 1 shows the range of differences in the antioxidant activity of the extracts/fractions from AST by the FRAP assay. The order of the antioxidant activity was as follows: WE (46.2 μmol Fe(II)/100 g) > 70% ethanol extract (29.4 μmol Fe(II)/100 g) > WT (20.5 μmol Fe(II)/100 g) > 95% ethanol extract (15.5 μmol Fe(II)/100 g) > EA (7.1 μmol Fe(II)/100 g) > BU (5.7 μmol Fe(II)/100 g) > PE (0.6 μmol Fe(II)/100 g). From the results, the WE was found to possess a significant reducing power, and among the WE fractions, the WT one has the highest reducing power. Thus, it was inferred that most of the antioxidant components from AST were soluble in water.

The correlation coefficient (R^2^) between the antioxidant activity and the total phenolic acid/flavonoid content of the extracts/fractions from AST was determined. The antioxidant activity and the total phenolic acid content (*R*^2^ = 0.8797) showed a better correlation than antioxidant activity and the total flavonoid content (*R*^2^ = 0.5555). Therefore, phenolic acid compounds are the dominant contributor to the antioxidant activity of AST. High phenolic acid content is thus an important factor in determining the antioxidant activity of this plant.

#### 2.2.2. Free radical (DPPH, ABTS) scavenging ability of the extracts/fractions from *Artemisia selegensis* Turcz

For evaluation of free radical scavenging properties of the extracts/fractions we have used two assays: the DPPH radical and the ABTS radical cation assay, and the results are shown in Table 2. The relatively stable organic radical DPPH has been widely used in the determination of antioxidant activity of different plant extracts [15]. From Table 2, the order of free radical scavenging ability of the extracts/fractions was found to be as follows, WE > 70% ethanol extract > WT > 95% ethanol extract > EA >BU > PE, which were consistent with FRAP assay. The WE possess a significant free radical scavenging ability (1081.7 μM ascorbic acid/100 g).

**Table 2:** Free radical scavenging abilityof the extracts and fractions from AST by the DPPH and ABTS assays.

Extracts/fractions	Free radical scavenging ability	
	DPPH ^a^	ABTS ^b^
WE	1081.7 ± 59.7	886.9 ± 3.3
70% ethanol extract	823.8 ± 33.4	583.3 ± 2.5
95% ethanol extract	477.7 ± 15.5	244.4 ± 2.5
PEE	15.8 ± 0.8	4.5 ± 0.3
EA	208.5 ± 4.6	99.6 ± 0.9
BU	143.6 ± 2.6	79.0 ± 1.1
WT	536.6 ± 16.9	362.1 ± 1.1

^a^ DPPH expressed in μM ascorbic acid/100 g dry weight of AST; ^b^ ABTS expressed in μM trolox /100 g dry weight of AST. Each value is the mean ± SD of triplicate measurements.

The correlation coefficient (R^2^) between the free radical (DPPH) scavenging ability and the total phenolic acid/flavonoid content of the extracts/fractions from AST was determined. The free radical scavenging ability and the total phenolic acid content of the extracts/fractions displayed a better correlation (*R*^2^ = 0.8515) than the free radical scavenging ability and total flavonoids content (*R*^2^ = 0.6660). The free radical scavenging ability of the extracts/fraction from AST was also determined using the ABTS radical cation. According to the data obtained (Table 2), the WE was again found to have the highest free radical scavenging ability (886.9 μM Trolox/100 g), and the order of ABTS results were consistent with that of DPPH and FRAP assays.

The correlation coefficient (R^2^) between the free radical (ABTS) scavenging ability and the total phenolic acid/flavonoid content of the extracts/fractions from AST was also determined. The free radical scavenging ability and the total flavonoids content (*R*^2^ = 0.5819) of the extracts/fractions has a lower correlation than free radical scavenging ability and total phenolic acid content (*R*^2^ = 0.8645). These results were consistent with the DPPH assay and FRAP assay data.

### 2.3. Antioxidant assay in vivo

D-(+)-Galactose has been shown to induce oxidative stress in both human and animal models, leading to the generation of potent reactive oxygen species (ROS), such as hydroxyl radical (OH˙). Plants are known to produce various antioxidant compounds to interact with ROS in order to survive [16]. In this study, we determined the antioxidant capacity of the WE from AST by monitoring its effect on antioxidant enzymes and the levels of malonaldehyde (MDA) in oxidatively stressed male mice.

Biological effects of ROS are controlled *in vivo* by a wide spectrum of enzymatic and non-enzymatic defense mechanisms, in particular superoxide dismutases (SOD), which catalyze dismutation of superoxide anions to hydrogen peroxide and catalase, which then converts H_2_O_2_ into molecular oxygen and water [17]. Their roles as protective enzymes are well known and have been investigated extensively both *in vivo* and in model systems [18].

Table 3 showed the activities of SOD in mouse serum. Results shown that SOD activity in all treatment groups exhibited significant increase compared to normal diet and D-(+)-galactose group (p < 0.01). From the results we can see that SOD activity of mice given high dose of WE group (nd+ D- gal+12 g WE/kg ) was highest, and even the low dose of WE group (nd+ D- gal+3 g WE/kg) has a higher SOD activity than the mice fed Vit C group. The results suggested that the WE possess a higher antioxidant activity than ascorbate.

**Table 3:** .Effect of the water extract from AST on SOD activity and lipid peroxidation content.

Groups	n	SOD activity (U/mL)	MDA content (nmol/mL)
nd+ D- gal	10	140.4 ± 19.4	8.4 ± 1.1
nd+ D- gal +0.4% Vit C	10	188.3 ± 34.7^**^	7.7 ± 1.2
nd+D- gal+12 g WE/kg	10	209.7 ± 44.6^**^	6.6 ± 1.3^*^
nd+ D- gal+6 g WE/kg	10	201.2 ± 45.4^**^	7.9 ± 1.1
nd+ D- gal+3 g WE/kg	10	192.2 ± 35.1^**^	8.2 ± 1.0

Data are shown as mean±S.D. ^*^P < 0.05 and ^**^P < 0.01 represent the significance of the difference from normal diet+ D-(+) Galactose group. Nd: normal diet.

Lipid peroxidation is an auto-catalytic, free-radical mediated, destructive process, whereby poly-unsaturated fatty acids in cell membranes undergo degradation to form lipid hydroperoxides [19]. These latter compounds then decompose to form a wide variety of products, including low molecular mass hydrocarbons, hydroxyl aldehydes, fatty acids, ketones and alkanals, in particular malonaldehyde (MDA) [20], Thus, reduction of MDA production would indicate inhibition of lipid peroxidation.

MDA level in these groups indicated the possibility of increased radical production and higher rate of lipid peroxidation in these mice. Table 3 reveals that MDA content in the serum of the mice treated with normal diet and D-gal group was highest. MDA contents of all the other groups decreased when compared with normal diet and D-gal treated group. As shown in Table 3, high dose of WE group significantly decrease the MDA level compared to normal diet and D-(+)-galactose group (*p* < 0.05), and the level was also lower than the group given Vit C.

In this study, the WE of AST exhibit significant inhibition of MDA production and cause a significant elevation of free radical scavenging enzyme activities such as SOD. Therefore, this research demonstrated that the extracts from AST have a higher antioxidant and free radical scavenging activities than ascorbate.

## 3. Experimental Section

### 3.1. Plant material

AST was collected from the Eight Diagrams Stata, Jiangsu, China in July. Collected plant material was dried in an oven at 50 °C for 48 h, The desiccated material was stored in a shaded and well-ventilated place and kept refrigerated in dark all-glass containers until extracted.

### 3.2. Chemicals

2,2’-Diphenyl-1-picrylhydrazyl (DPPH), 6-Hydroxy-2,5,7,8-tetramethylchroman-2-carboxylic acid (Trolox), Folin-Ciocalteu’s phenol reagent, 2,2’-Azino-bis(3-ethylbenzothiazoline-6-sulfonic acid) diammonium salt (ABTS), and rutin were purchased from Sigma-Aldrich (St. Louis, MO). 2,4,6-tri(2-Pyridyl)-s-triazine (TPTZ) was purchased from Fluka Chemie AG (Buchs, Switzerland). All other chemicals and reagents were of analytical grade, and they were used as received.

### 3.3. Extraction and fractionation of antioxidants

The dried AST sample was first ground to fine powder. For 70% ethanol extraction, the powder (100 g) was immersed in 75% ethanol (1,000 mL) for 1 h and then refluxed for 1.5 h. The extract was filtered through cloth and the residue was re-extracted under the same conditions. The combined solution was concentrated by rotary evaporation at 50 °C under vacuum; the resulting residue was moved to a vacuum oven at 40 °C and dried for 48 h to give a solid extract (7.2664 g).

For 95% ethanol extraction, the powder (100 g) was treated using the same procedures as for the 70% ethanol extraction, producing 3.9823 g of a solid extract.

For water extraction, the fine powder (100 g) was immersed in water (1,000 mL) for 1 h and boiled for 1.5 h. The extract was filtered through cloth and the residue was re-extracted under the same conditions. Following the same concentration procedures as those used for the 70% ethanol extraction, the extract was concentrated and dried to produce 11.1046 g of solid extract.

Because the water extract has a high antioxidant activity, it was further fractionated with different solvents. Briefly, the aqueous extract (combined from two extractions of 100 g each) was successively extracted with 100 mL of petroleum ether, ethyl acetate and *n*-butanol saturated with water. Solvents from each fraction and the remaining water were removed with a rotary evaporator at 40-50 °C under vacuum, and the residues were moved into a vacuum oven at 40 °C for 48 h. After removal all of the solvents, four fractions were obtained, including the petroleum ether (PE), ethyl acetate (EA), *n*-butanol (BU) and water (WT) fractions, with the solid residues being 0.0390 g, 0.5957 g, 0.8234 g and 9.1839 g, respectively.

The water extract, 70% ethanol extract, 95% ethanol extract and four subfractions obtained from further extraction of aqueous extract were dissolved in deionized water or 95% ethanol. The resulting solutions (containing various extracts) were used to evaluate the antioxidant activities of AST by the POV, ABTS, DPPH and FRAP assays. The water extract was also used to estimate the antioxidant activity of AST *in vivo* by measuring its ability to reduce oxidative stress in mice.

### 3.4. Determination of total phenolic acids

The total phenolic acids were estimated according to the Folin-Ciocalteu method [21]. To sample (100 μL) was added undiluted Folin-Ciocalteu-reagent (2.0 N, 200 μL). After 2 min, 20% (w/v) aqueous Na_2_CO_3_ (700 μL) were added, and the volume was made up to 4.0 mL with “nanopure” water. The control contained all the reaction reagents except an extract of interest. The solution was incubated at room temperature (25 °C) in the dark for 2 h. The absorbance was measured at 760 nm and compared to gallic acid equivalents, using a gallic acid (0–0.6 mg/mL) standard curve. Additional dilution was done if the absorbance value measured was over the linear range of the standard curve. The results were expressed as mg gallic acid equivalents (GAE)/100 g dry weight of plant material. All the measurements were taken in triplicate and the mean values were calculated.

### 3.5. Determination of flavonoids

The AlCl_3_ method [22] was adapted for the purpose of determining the total flavonoid content of AST extracts. Predetermined quantities of extracts were put into 50 mL volumetric flasks and dissolved with suitable solvents and made up to 50 mL. Aliquots (2.0 mL) were transferred into 25 mL volumetric flasks. 5% (g/v) NaNO_2_ (1.0 mL) was added, and the mixture were allowed to react for 6 min. Then, 10% AlCl_3_ (g/v, 1.0 mL) was added, and the mixture were allowed to react for another 6 min. Afterwards, 10% (g/v) NaOH (10 mL) was added, and thei volumes were made up to 25 mL with 50% (v/v) ethanol. The solution was incubated at room temperature (25 °C) for 15 min and absorbance recorded at 510 nm against a blank (no extract). The amount of flavonoids was calculated as a rutin equivalent from the calibration curve of rutin standard solutions, and expressed as mg rutin/100 g dry weight of plant material.

### 3.6. Determination of antioxidant capacity in vitro

#### 3.6.1. Ferric Reducing Antioxidant Power (FRAP) assay

The total antioxidant potential of each extract was determined using the ferric reducing antioxidant power (FRAP) assay of Szeto and Chu [23] with slight modifications. Briefly, the FRAP reagent was prepared from 300 mM acetate buffer (pH 3.6), 10 mM TPTZ solution in 40 mM HCL and 20 mM iron (III) chloride solution in proportions of 10:1:1 (v/v), respectively. The FRAP reagent was prepared fresh daily and was warmed to 37 °C in a water bath before use. AST extracts (50 μL) were added to FRAP reagent (3 mL). After 4 min, the absorbance of the colored product (ferrous tripyridyltriazine complex) was then recorded at 593 nm. The standard curve was constructed using iron (II) sulfate solution (0–3,000 μM), and the results were expressed as μmol Fe (II)/100 g dry weight of plant material. Additional dilution was made if the FRAP value measured was over the liner range of the standard curve. All the measurements were taken in triplicate and the mean values were calculated.

#### 3.6.2. DPPH radical scavenging assay

The DPPH radical scavenging activity of AST extracts was determined using the method of Von Kaji, Inukai and Maiguma [24] with some modifications. Briefly, the tested samples (150 μL) were allowed to react with 1 × 10^-4^ M DPPH solution (3 mL) in absolute alcohol. The decrease in absorbance at 517 nm was determined after 16 min for all samples. The absorbance of 3 mL DPPH radical solution as control was measured in each assay. The standard curve was liner between 0–680 μM ascorbic acid. Results are expressed in μM ascorbic acid/100 g dry weight of plant material. Additional dilution was needed if the DPPH value measured was over the linear range of the standard curve. All determinations were performed in triplicate. The percentage inhibition of the DPPH radical by the samples was calculated according to the formula of Yen and Duh [25]:

% inhibition = [(*A*c_(0)_-*A*_A(t)_)/ *A*c_(0)_] × 100 (1)

where *A*c_(0)_ is the absorbance of the control at *t* = 0 min; and *A*_A(t)_ is the absorbance of the antioxidant at *t* = 16 min.

#### 3.6.3. ABTS radical cation assay

The free radical scavenging activity of AST extracts was determined by the ABTS radical cation decolorization assay [26] with some modifications. ABTS radical cation (ABTS^+^) was produced by hydrogen peroxide solution of the acetic acid-sodium acetate buffer (30 mM, pH 3.6). The solution was prepared as follows: sodium acetate (2.46 g) was dissolved in deionized water (1,000 mL, final concentration: 30 mM). Glacial acetic acid (1.705 mL) was diluted to 1,000 mL with deionized water (final concentration: 30 mM). The sodium acetate solution (75 mL) was mixed with the acetic acid solution (925 mL) under a pH meter; the pH of acetic acid-sodium acetate buffer was 3.6. Then H_2_O_2_ solution (278 μL, 35%) was diluted to 1,000 mL with the buffer solution (final concentration: 2 mM). Then ABTS (0.550 g) was dissolved in prepared solution (100 mL, final concentration: 10 mM). After 1 h of incubation at room temperature, the characteristic blue-green color of ABTS**^.^** ^+^ appeared. The colored reagent was stable for at least 6 months at 4 °C.

For the study of AST extracts, the ABTS**^.^** ^+^ solution was diluted with the acetic acid-sodium acetate buffer (30 mM, pH 3.6 ) to an absorbance of 0.70 (±0.02) at 734 nm and equilibrated at 30 °C. The absorbance of ABTS**^.^** ^+^ without antioxidant as control measured in each time (*A*_0_). After addition of 3.0 mL of diluted ABTS**^.^** ^+^ solution (*A*_734nm_ = 0.700 ± 0.020) to 150 μL of AST extracts or Trolox standards (final concentration 0–520 μM) in absolute alcohol the solutions were vortex mixed for exactly 30 s; the absorbance at 734 nm was taken at 30 °C exactly 6 min after initial mixing (*A*_t_). Appropriate solvent blanks were run in each assay. All determinations were carried out at least three times, on each occasion and at each separate concentration of the standard and samples. The percentage inhibition of absorbance at 734 nm was calculated using the following formula:

% inhibition = [*A*_0_-(*A*t-*B*)]/*A*_0_× 100 (2)

where *A*_0_ is the absorbance of the control at *t* = 0 min; *A*t is the absorbance of the antioxidant at *t* = 6 min; and *B* is the absorbance of the blank solutions of sample.

### 3.7. Antioxidant assay in vitro

D-(+)-Galactose (D-gal) has been shown to induce oxidative stress in animal models, leading to the generation of potent reactive oxygen species (ROS), such as hydroxyl radical (OH^.^). Oxidative stress results when generation of reactive oxygen and activity of the antioxidant defenses are unbalanced. The increase in ROS could be due to their excessive production and/or decreased destruction. Cells exposed to severe oxidative stress may suffer degeneration of DNA, membrane lipids and protein and enzymes, leading to various pathological conditions. We determined the antioxidant capacity of the WE from AST by monitoring its effect on antioxidant enzymes and the levels of malonaldehyde (MDA) in oxidatively stressed male mice.

#### 3.7.1. Animals and diets

Fifty male adult mice, approximately 3 months old, weighing in the range of 18–22 g were selected for the study. All the mice were healthy and not infected with virus or bacteria. The mice were randomly divided into five groups of ten mice each. Group I was given D-gal and normal laboratory diet; Group II was given D-gal, 0.4% ascorbate (Vit C) and normal laboratory diet; Group III was given D-gal, WE from AST at a dose of 12 g/kg per day and normal laboratory diet; Group IV was given D-gal, the WE at a dose of 6 g/kg per day and normal laboratory diet; Group V was given D-gal, the WE at a dose of 3 g/kg per day and normal laboratory diet. The WE was administered orally by gastric intubation, D-gal was administered by hypodermic injection at a dose of 1.2 g/kg per day. Animals were kept in cages in a room that was maintained at 28 °C and water was given *ad libitum*. The duration of the experiment was one month. Body weight record was kept weekly. At the end of the experimental period, the mice were deprived of food but not water overnight. Blood was collected by ophthalmic vein and drawn into centrifuge tubes containing heparin for serum collection. At the end of the experiment, mice were sacrificed by euthanasia.

#### 3.7.2. Preparation of serum

The blood of mice was collected in centrifuge tubes containing heparin, followed by centrifugation at 3,000 rpm for 15 min (37 °C). The supernatant was then used for measurement of superoxide dismutase (SOD) activity and the contents of malonaldehyde (MDA).

#### 3.7.3. Determination of antioxidant enzyme activities and lipid peroxidation

SOD activity and lipid peroxidation were determined with the use of kits obtained from Nanjing Jiancheng Bioengineering Institute (Cat. No. A001-1 and No. A003, respectively). The determination principle of SOD kit was that the superoxide anion (O_2_^-^) generated from the reaction of xanthine and xanthine oxidase and then the O_2_^-^ oxidized the hydroxylamine, which generated the nitrite. Nitrite could be presented purplish red by the effect of chromogenic agent. Then the absorbance was taken at 550 nm by spectrophotometer. When the samples are containing SOD, the superoxide anion radicals could be distinctively inhibited and as a result, the nitrite was reduced. The SOD activity of samples can be measured according to the formula.

The assessment of the extent of lipid peroxidation relied on individual determinations of MDA contents in serum. MDA is an end product of peroxidation and its accumulation in tissues is the indication of the extent of lipid peroxidation [27]. The determination principle of MDA kit present as follows: MDA was the degradation product of lipid peroxidation and could be reacted with thiobarbituric acid and then a red product with a maximum absorption at 532 nm was generated.

### 3.8. Statistical analysis of data

Data were presented as means ± SD of at least triplicate experiments. Analysis of variance was performed on the data obtained. Significance of differences between means was determined by least significant differences (LSD) at *P* ≤ 0.05.

## 4. Conclusions

*In vitro* antioxidant studies, results of FRAP, DPPH and ABTS assays showed that AST extracts possess not only the antioxidant activities, but also potent free radical scavenger capability. WE was found to have the highest antioxidant activity, and among the subfractions of WE, the WT one has the highest antioxidant activity. Thus, it was deduced that most of the antioxidant components from AST were soluble in the water.

We compared the correlations between the antioxidant activity and the total phenolic acid/flavonoid content of the extracts/fractions from AST, and found that antioxidant activity and total phenolic acid content showed a better correlation than antioxidant activity and flavonoid content, indicating that phenolic acid compounds are dominant contributors to the antioxidant activity of AST.

We also determined the antioxidant activity of the WE *in vivo* by monitoring its effect on antioxidant enzymes (SOD) and the levels of malonaldehyde (MDA) in the male mice with induced oxidative stress. The WE of AST exhibits significant inhibition of MDA production and cause a significant elevation of free radical scavenging enzyme activities. Therefore, this research demonstrated that the WE from AST has strong antioxidant and free radical scavenging capabilities. Therefore, we propose that AST could be potentially used as rich source of natural antioxidants.
